# CLIMATE CHANGE: Challenges of Predicting Wildfire Activity

**DOI:** 10.1289/ehp.117-a293

**Published:** 2009-07

**Authors:** Carol Potera

**Affiliations:** Montana-based **Carol Potera** has written for *EHP* since 1996. She also writes for *Microbe, Genetic Engineering News, and the American Journal of Nursing*

An increase in wildfire activity is one of several effects predicted to arise in some areas as a result of climate change. Two new studies suggest, however, that wildfires are more complex—and their future prevalence less predictable—than is commonly assumed. “We hear a lot that the entire planet will be burning in the future, but there’s not good evidence to back this up,” says Max Moritz, co-director of the Center for Fire Research and Outreach at the University of California, Berkeley.

Moritz and his colleagues designed a new pyrogeography model to predict wildfire activity out to 2099. Their novel “ecosystem niche” model treats fire as an organism within an ecosystem and characterizes its “habitat requirements,” with flammable vegetation serving as the consumable resource and fire-conducive weather patterns and climate—such as hot, dry winds and temperatures—constituting the environmental conditions. “Our results challenge the simplistic view that climate change will cause more fires everywhere,” Moritz says.

The model, described in the 8 April 2009 edition of *PLoS ONE*, predicted that wildfires could increase in the western United States and Tibetan plateau but decrease in northeast China and central Africa. The leading single predictor of wildfires was the amount of vegetation available to burn, followed by hot, dry conditions, yet neither alone ensures fire. For instance, rainforests contain dense vegetation, but high moisture levels constrain fire activity, which could explain why most tropical evergreen forests of the central Amazon and the Congo have stayed relatively fire-free even during anomalously dry periods. Deserts, on the other hand, are hot and dry but lack sufficient vegetation to support fires.

Vegetation also proved a key ingredient in ancient wildfires that burned in Alaska’s Brooks Range some 15,000 years ago, suggest Philip Higuera and colleagues in the May 2009 issue of *Ecological Monographs*. They examined sediment cores collected at various depths from lake bottoms to assess changes in pollen and other plant materials that grew during certain periods. They used carbon-14 dating to determine the age of charcoal found in the cores—an indicator that a fire had occurred.

Higuera’s team discovered that the impact of climate change on fire occurrence was often mediated by changes in vegetation, sometimes in ways that seemed counter intuitive. The climate transitioned from cool and dry to warm and dry about 10,500 years ago, yet fire frequency declined sharply over the course of about 1,000 years. This was likely due to a climate-induced shift in vegetation from flammable shrubs to more fire-resistant deciduous trees. In contrast, the team observed an increase in fire frequency about 5,000 years ago despite the onset of cooler temperatures and higher moisture levels. The rise of dense, highly flammable coniferous forests probably fueled these fires.

The importance of vegetation in facilitating or retarding fire is well known by fire scientists, says Higuera, an ecologic research fellow in the Department of Earth Sciences at Montana State University, Bozeman. What these two new studies offer, he says, are the large spatial and temporal scales at which they were conducted. “At these scales, the influence of vegetation is more apparent than it is at the smaller scales on which earlier studies have focused,” he explains, “and we can make statements about the impacts of climate change on fire regimes over large spatial and temporal scales.”

Higuera’s findings from ancient times may apply to today’s changing climate. Several studies have predicted that in Alaska, where temperatures are climbing faster than at lower latitudes, conifers will burn and be replaced by deciduous trees such as aspen and birch, typically the first species to recolonize an area after a fire. “Our study suggests there will be a decrease in the probability of fire with a transition to [less flammable] deciduous forests,” says Higuera. He adds that such transitions have happened over multiple centuries in the past, but it is possible they could happen more quickly in the future because of human impact.

“The patterns found by Higuera are striking, but it has yet to be shown that similar patterns are seen in places other than central Alaska,” says University of Oregon geography professor Dan Gavin, who with Higuera coauthored a report in the October 2008 issue of *Nature Geoscience* on global biomass burning over the past two millennia. In many other regions, paleoecologic studies have shown that fires increased as the climate became warmer and drier, and decreased as it became cooler and wetter.

The interactions between climate, geography, ecosystems, and wildfires have major implications for public health. Beyond the obvious air quality issues associated with smoke, a study by Teresa C. Wegesser and colleagues in the June 2009 issue of *EHP* indicated that particulate matter produced during California’s 2008 wildfire season was more toxic to the lungs than particles collected from ambient air.

Wildfires can also have indirect health consequences. For instance, in work published online 28 May 2009 ahead of print in *Zoonoses and Public Health*, veterinary biologist Hume E. Field of the Australian Biosecurity Cooperative Research Centre for Emerging Infectious Diseases noted that recent deforestation and fires in Australia and Asia may have disrupted habitat for fruit bats (genus *Pteropus*). Field and others hypothesize that fire-related deforestation and other ecologic forces have driven fruit bats to seek food closer to towns, where they spread fatal Hendra and Nipah viruses to farm animals and people. “These more subtle health effects of fire are hardly looked at,” says Moritz.

## Figures and Tables

**Figure:**
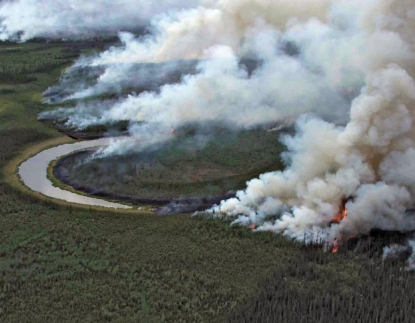
The forest burns along the Sheenjek River near Fort Yukon, Alaska, June 2005.

